# Segment-Specific Analysis of Carotid Intima-Media Thickness and Its Association with Cardiovascular Risk Factors in a Large Healthy Cohort

**DOI:** 10.3390/jcm14061918

**Published:** 2025-03-12

**Authors:** Hyo-In Choi, Yun Tae Kim, Jeong Gyu Kang, Yuna Kim, Jong-Young Lee, Ki-Chul Sung

**Affiliations:** 1 Division of Cardiology, Department of Internal Medicine, Kangbuk Samsung Hospital, Sungkyunkwan University School of Medicine, Seoul 04514, Republic of Korea; jyleeheart.lee@samsung.com (J.-Y.L.); kcmd.sung@samsung.com (K.-C.S.); 2 Division of Biostatistics, Department of Academic Research, Kangbuk Samsung Hospital, Sungkyunkwan University School of Medicine, Seoul 04514, Republic of Korea; yt9771.kim@samsung.com; 3 Center for Cohort Studies, Total Healthcare Center, Kangbuk Samsung Hospital, Sungkyunkwan University School of Medicine, Seoul 04514, Republic of Korea; jg1980.kang@samsung.com; 4 Total Healthcare Center, Kangbuk Samsung Hospital, Sungkyunkwan University School of Medicine, Suwon 16419, Republic of Korea; starrynkim@gmail.com

**Keywords:** carotid arteries, vascular intima, risk factors, cardiovascular disease, atherosclerosis

## Abstract

**Background:** Carotid intima-media thickness (IMT) is a noninvasive surrogate marker of subclinical atherosclerosis and cardiovascular disease risk. This study explored IMT distribution across three carotid artery segments in a large cohort of healthy individuals and identified the key factors associated with increased IMT. **Methods:** This study utilized data from the Kangbuk Samsung Health Study, a cohort of South Korean adults aged ≥ 18 years who underwent comprehensive annual or biennial health examinations. The analysis included 86,351 healthy individuals, excluding those with known carotid disease. IMT was measured using high-resolution B-mode ultrasonography across the three segments: common carotid artery (CCA), carotid bulb, and internal carotid artery (ICA). An increased IMT was defined as a measurement of ≥1.5 mm in any segment. Multivariable linear regression analyses were conducted to identify independent predictors of increased IMT. **Results:** The study population had a mean age of 46.7 years and was predominantly male (69.7%). The prevalence of thickened IMT was the highest in the carotid bulb, followed by the ICA and CCA. IMT increased progressively with age and was higher in males across all segments, with the disparity becoming more pronounced after 65 years of age. The carotid bulb displayed the largest absolute IMT values, whereas the ICA exhibited a sharper age-related increment. Increased CCA IMT was strongly linked to hypertension (beta, 0.11; *p* < 0.001) and diabetes mellitus (beta, 0.12; *p* < 0.001). Both CCA and ICA IMT showed a weak but significant association with dyslipidemia (beta, 0.03; *p* < 0.001). **Conclusions:** The IMT distribution and its determinants vary across carotid segments. CCA is a robust marker of systemic vascular health, whereas the carotid bulb is the most sensitive marker for detecting early atherosclerotic changes. This study provides novel insights into segment-specific IMT patterns and their association with cardiovascular risk factors in a large, healthy Asian population.

## 1. Introduction

Carotid intima-media thickness (IMT) is a well-established noninvasive marker of subclinical atherosclerosis [1,2] and a predictor of cardiovascular (CV) disease risk [3,4]. Increased IMT has been consistently associated with major CV events [5,6], including myocardial infarction [7] and stroke [8] in various populations [9,10]. Its widespread use as a risk marker is supported by its practicality, affordability, reliability, and safety, making it an essential tool for CV risk assessment. The 2022 guidelines of the Korean Society of Hypertension (HTN) recognize the potential utility of carotid plaque assessment in predicting CV risk, particularly in high-risk individuals [11]. However, they emphasized the limitations of carotid IMT measurement, including the lack of standardized methodologies and insufficient evidence supporting its routine use in screening for the prediction of cardiovascular events. Similarly, the 2017 European Society of Cardiology guidelines suggest that carotid IMT measurement may be considered for high-risk individuals but do not recommend routine use due to similar concerns [12]. The 2013 American College of Cardiology/American Heart Association guidelines do not recommend carotid IMT measurement for routine practice, citing its limited incremental predictive value over traditional risk factors [13] as it adds only a modest predictive value to traditional risk factors [1]. These guidelines collectively highlight the need for improved standardization and more extensive data to enhance the clinical applicability of carotid IMT measurement.

Such limitations are particularly relevant in Asian populations where data on carotid IMT are relatively limited. Although earlier studies linked carotid IMT to coronary heart disease and stroke in the Chinese population, these findings predate the widespread use of modern lipid-lowering and antihypertensive therapies [14]. Although limited by a small sample size, more recent research has established reference intervals for carotid IMT in a multi-ethnic Asian population, highlighting significant associations with cardiometabolic risk factors and underscoring the importance of considering ethnic-specific variations in CV risk assessment [15]. Additionally, many large-scale studies do not provide detailed segmentation analyses, such as comparisons between the common carotid artery (CCA), internal carotid artery (ICA), and carotid bulb, which could offer deeper insights into regional variations in atherosclerosis [16]. To address these gaps, this study analyzed a large cohort of healthy South Korean adults by using recent carotid ultrasonography data. It examined segmental IMT distributions and their associations with metabolic risk factors, offering updated insights based on contemporary measurement techniques.

## 2. Materials and Methods

### 2.1. Study Design and Population

This study utilized data from the Kangbuk Samsung Health Study, an ongoing cohort of South Korean adults aged ≥18 years who underwent comprehensive health screenings at the Kangbuk Samsung Hospital Health Screening Centers in Seoul and Suwon [17]. Participants with established carotid artery disease (>50% carotid artery stenosis, based on imaging data) and individuals with missing data or poor-quality carotid ultrasound images, characterized by low resolution, shadowing, or absence of key segments, as assessed by two independent cardiologists, were excluded to ensure the accuracy of the analysis. This study was approved by the Institutional Review Board of Kangbuk Samsung Hospital and conducted in accordance with the Declaration of Helsinki. As this was a retrospective study using anonymized health-screening data, the requirement for written informed consent was waived.

### 2.2. Carotid Ultrasonography and IMT Measurement

Carotid ultrasonography was performed using high-resolution B-mode imaging with a GE LOGIQ E9 ultrasound system equipped with a 9 L-D linear array transducer (GE Healthcare, Milwaukee, WI, USA), ensuring precision in vascular assessment [18]. IMT measurements were obtained for three carotid artery segments: CCA, carotid bulb, and ICA. The CCA was assessed 1 cm proximal to the carotid bifurcation, the carotid bulb at its widest part, and the ICA within the first 1 cm distal to the bifurcation. Measurements of the far-wall IMT at each segment were performed following the American Society of Echocardiography (ASE) guidelines for carotid ultrasonography [19]. Automated edge detection software (GE Healthcare, Milwaukee, WI, USA) was primarily used for measurements, with experienced sonographers manually adjusting segments as needed in cases of increased thickness or suspected lesions. The equipment used in this study operated on various software versions (ranging from R2 to R6), but the Auto IMT Measurement function has remained consistent since R1. IMT values were classified as normal (<1.5 mm) or thickened (≥1.5 mm), a threshold that also encompasses carotid plaques, as defined by the Mannheim Consensus criteria. Quality control involved periodic image reviews by four cardiologists specializing in imaging to ensure measurement accuracy. We assessed the repeatability and reproducibility of IMT measurements using intra-class correlation coefficient (ICC), coefficient of variation (CV), and Bland–Altman analysis with the full dataset, including multiple follow-up measurements from the same individuals. Despite minor temporal variations, these analyses confirm high measurement reliability. Full details are provided in Supplementary Table S1.

### 2.3. Variables Assessment

Key variables included demographic factors, medical history (e.g., hypertension (HTN), diabetes mellitus (DM), and dyslipidemia), lifestyle behaviors (e.g., smoking status, physical activity, and sedentary behavior), and laboratory data (e.g., lipid profile, renal function, and natriuretic peptide levels). HTN was defined as a systolic blood pressure ≥140 mmHg, diastolic blood pressure ≥90 mmHg, or current use of antihypertensive medication. DM was identified based on self-reported history, use of antidiabetic medications, or laboratory criteria of fasting serum glucose level ≥126 mg/dL or hemoglobin A1c (HbA1c) level ≥6.5%. Dyslipidemia was defined based on self-reported history, the use of lipid-lowering medications, or biochemical criteria, including total cholesterol ≥ 240 mg/dL, LDL cholesterol > 160 mg/dL, HDL cholesterol < 40 mg/dL in men or < 50 mg/dL in women, or triglycerides (TGs) ≥ 200 mg/dL. Medication use was assessed using self-report questionnaires. Anthropometric measurements, such as height and weight, were recorded with participants wearing light clothing and no shoes. Body mass index (BMI) was calculated as the weight in kilograms divided by the height in meters squared. Blood pressure was measured using an automated oscillometric device (53,000; Welch Allyn, New York, NY, USA) by professional nurses. Three consecutive measurements were taken from the same arm, with a 30 s interval between each measurement. The average of the second and third readings was used for analysis to ensure accuracy. Laboratory tests performed after overnight fasting included lipid profiles (total cholesterol, low-density lipoprotein cholesterol (LDL-C), high-density lipoprotein cholesterol (HDL-C), and triglycerides (TGs)), glucose levels, high-sensitivity C-reactive protein (hsCRP), N-terminal pro-B-type natriuretic peptide (NT-proBNP), and insulin resistance, assessed using the homeostatic model assessment of insulin resistance (HOMA-IR). Physical activity and sedentary behavior were evaluated using a validated self-reported questionnaire that categorized activity levels by frequency and intensity.

### 2.4. Statistical Analysis

The baseline characteristics of the study population are summarized as mean ± standard deviation (SD) for continuous variables and as frequencies with percentages for categorical variables. Where appropriate, medians and interquartile ranges were also reported for continuous variables. Comparisons between individuals with normal and thickened IMT were conducted using *t*-tests for continuous variables and chi-squared tests for categorical variables. The distribution of carotid IMT by sex and five-year age intervals was visualized using mean values and standard errors represented using error bars. Multivariable linear and logistic regression analyses were used to evaluate the association between carotid IMT and cardiovascular risk factors. Linear regression results were presented as regression coefficients with 95% confidence intervals (CIs), while logistic regression findings were expressed as odds ratios (ORs) with 95% CIs. Sequential adjustment models were applied to control for potential confounders: Crude (unadjusted); Model 1 (adjusted for age and sex); Model 2 (additional adjustments for BMI, NT-proBNP, eGFR, and HOMA-IR); and Model 3 (further adjustment for physical activity). A two-tailed *p*-value <0.05 was considered statistically significant. Sensitivity analysis was performed to validate the robustness of the results. All analyses were conducted using R statistical software (version 4.2.0).

## 3. Results

### 3.1. Baseline Characteristics

This study analyzed 87,523 participants with a mean age of 46.7 years (SD: 9.7), of whom 69.7% were male. Table 1 presents the baseline characteristics of the participants with and without thickened IMT (≥1.5 mm) across the CCA, carotid bulb, and ICA. Thickened IMT was most frequently observed in the carotid bulb (11.9%), followed by the ICA (1.7%) and CCA (1.2%). Participants with thickened IMT were older in all segments (*p* < 0.001). Males had higher IMT values across all segments, most notably in the ICA, where 82.6% of the participants with thickened IMT were male. Blood pressure was significantly higher in participants with thickened IMT, particularly in the CCA segment (*p* < 0.001). Similarly, in the ICA, participants with thickened IMT had higher fasting glucose and HbA1c levels (*p* < 0.001). The carotid bulb showed significantly higher rates of HTN and sedentary behavior (*p* < 0.001). Vigorous physical activity levels were lower in participants with thickened IMT across all segments (*p* < 0.001). Alcohol consumption was also higher among participants with thickened IMT in all the segments. Although the differences in BMI and waist circumference between groups reached statistical significance (*p* < 0.05), the absolute differences were small (~0.2–1.0 kg/m^2^), which may limit their clinical relevance. However, the differences in systolic blood pressure and fasting glucose were larger and may have greater implications for cardiovascular risk.

### 3.2. Age, Sex, and Segmental Variations in Carotid IMT

**Figure 1:**
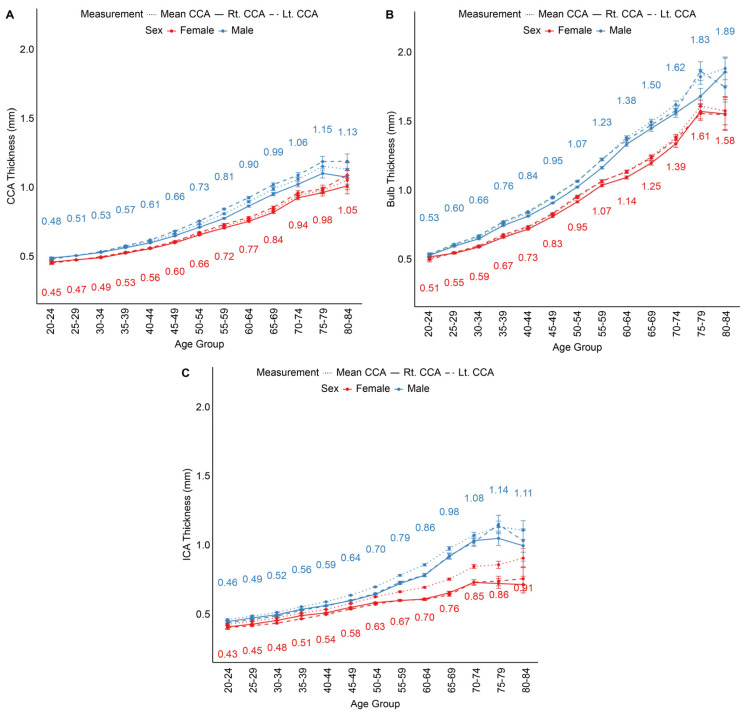
Age- and sex-stratified intima-media thickness (IMT) measurements. (**A**) Common carotid artery (CCA); (**B**) carotid bulb; (**C**) internal carotid artery (ICA). Error bars represent standard errors of the mean.

IMT was analyzed in 5-year age intervals from 20 to 85 years (Figure 1) and stratified by sex. Across all measured segments—the CCA, carotid bulb, and ICA—IMT progressively increased with advancing age, with the increase becoming more pronounced after 60 years of age. Males consistently exhibited higher IMT values than females across all segments, and this difference was more prominent in older age groups, particularly in the ICA.

The carotid bulb had the highest IMT values across all age groups, likely due to the effects of turbulent flow at the bifurcation. By the age of 65–69 years, the mean IMT in the carotid bulb of males exceeded 1.5 mm, with some individuals surpassing this threshold at earlier ages. In the oldest age group, the mean value reached 1.89 mm. The ICA showed higher IMT values than the CCA, with IMT in males exceeding 1.0 mm in the 55–59 age group and reaching approximately 1.29 mm by 80–84 years. In contrast, the mean IMT in the CCA remained below 1.2 mm across all age groups, showing a gradual and consistent increase over time.

### 3.3. Associations of HTN, DM, and Dyslipidemia with Carotid IMT

Regression analyses were conducted to examine the associations between HTN, DM, and dyslipidemia and carotid IMT across segments, focusing on continuous IMT values (Table 2) and thickened IMT (≥1.5 mm) (Table 3). In the CCA, HTN had a beta value of 0.11 in the crude model (*p* < 0.001), which remained significant after full adjustment in Model 3 (beta = 0.03, *p* < 0.001). Similarly, DM in the CCA showed a beta value of 0.12 in the crude model and 0.03 in Model 3 (*p* < 0.001). Dyslipidemia exhibited smaller associations in the CCA, with a beta value of 0.03 in the crude model and 0.01 in Model 3, both statistically significant. In the carotid bulb, HTN and DM maintained significant associations after adjustment, with HTN showing a beta value of 0.05 in Model 3 (*p* < 0.001), and DM with a beta value of 0.03 (*p* = 0.014). In the ICA, the association with dyslipidemia was weak but statistically significant (beta, 0.03; *p* < 0.001), similar to that observed in the CCA segment. While the beta coefficients for dyslipidemia and IMT were statistically significant (*p* < 0.001), their absolute effect sizes were small (β = 0.01–0.03). These findings suggest that, while dyslipidemia may contribute to IMT progression, its direct impact on carotid thickening is relatively modest compared to hypertension and diabetes mellitus.

For thickened IMT (≥1.5 mm), HTN had the highest odds ratio in the CCA crude model (OR = 4.04, *p* < 0.001), which decreased to 1.60 in Model 3 (*p* = 0.011). DM was independently associated with thickened IMT across all segments, with the strongest association being observed in the carotid bulb (OR = 2.13, Model 3; *p* < 0.001). Dyslipidemia showed significant associations with thickened IMT in the CCA (OR = 1.59, Model 3; *p* = 0.011), the carotid bulb (OR = 1.25, Model 3; *p* = 0.003), and the ICA (OR = 1.42, Model 3; *p* = 0.038).

### 3.4. Reference Distribution of Carotid IMT by Age and Sex

**Figure 2:**
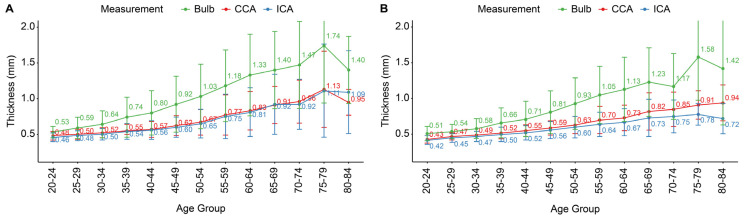
Age-specific normal carotid intima-media thickness (IMT) values in individuals without a history of hypertension, diabetes mellitus, or dyslipidemia, measured across three carotid segments: CCA (common carotid artery), ICA (internal carotid artery), and bulb. (**A**) Males. (**B**) Females. Error bars represent standard deviations.

Carotid IMT was assessed in individuals without a history of DM, HTN, or dyslipidemia. Table 4 and Table 5 summarize the mean (SD) and median (IQR) IMT values across age groups and carotid segments for males and females, respectively. Figure 2 illustrates the age-specific normal IMT values for males (A) and females (B). In both sexes, IMT in the CCA, carotid bulb, and ICA increased progressively with age. In males, the mean CCA IMT ranged from 0.48 mm in the 20–24 age group to 1.13 mm in the 75–79 age group. In females, it ranged from 0.43 mm to 0.94 mm over the same age range. The carotid bulb exhibited the greatest variability, especially in older age groups. At CCA IMT levels of 0.8–0.9 mm, which are commonly used as clinical thresholds, the corresponding mean ICA IMT levels were approximately 0.8–0.9 mm in males and 0.7–0.8 mm in females. However, the carotid bulb was noticeably thicker at the same CCA thickness, with mean IMT levels of 1.2–1.4 mm in males and 1.1–1.3 mm in females. The mean CCA and ICA IMT exceeded 0.8 mm in both sexes starting at the age of 60 years. In this population without HTN, DM, or dyslipidemia, the differences in IMT between males and females were less pronounced compared to those observed in the overall population.

## 4. Discussion

This study provides a detailed characterization of carotid IMT in a large cohort of South Korean adults, focusing on segment-specific differences, associations with CV risk factors, and age- and sex-specific reference values in individuals without major CV risk factors. As one of the largest and most recent studies on carotid IMT in Asian populations, these findings serve as a resource for future research and offer valuable insights into the regional epidemiology of subclinical atherosclerosis.

### 4.1. Segmental Characteristics of IMT

Distinct segmental differences in carotid IMT were observed across the CCA, carotid bulb, and ICA. Thickened IMT was most prevalent in the carotid bulb (11.9%), followed by the ICA (1.7%), and CCA (1.2%). Interestingly, LDL-C levels were lower in participants with thickened IMT, which may be attributed to the high prevalence of lipid-lowering therapy in this population. Statin therapy has been shown to reduce LDL-C levels while also slowing the progression of carotid atherosclerosis, which may have led to this inverse association. The lower TG levels observed in the bulb-IMT <1.5 mm group may be indicative of differences in metabolic status, dietary habits, or physical activity levels, which warrant further investigation. CCA exhibited the lowest and most stable IMT values, aligning with its role as a reference site in clinical practice [20]. Its relatively simple flow dynamics and uniform arterial wall structure make it the preferred site for IMT measurement in most studies and guidelines. However, these characteristics may also limit its ability to capture advanced atherosclerotic changes compared to more complex regions, such as the carotid bulb or ICA [21]. In contrast, the carotid bulb naturally exhibits thicker IMT values owing to the turbulent flow and its anatomical structure. For example, normal IMT values in the bulb are typically higher than in the CCA [22], with previous studies suggesting averages around 1.1 mm in healthy individuals [23]. Despite this, there is no widely accepted segment-specific threshold for defining a thickened IMT in the bulb. In our study, the prevalence of thickened IMT (≥1.5 mm) was the highest in the bulb, indicating that generalized thresholds may overestimate abnormal findings in this segment. This highlights the importance of interpreting IMT measurements in the context of segment-specific characteristics. The ICA demonstrated intermediate IMT values, with a notable divergence between males and females in the older age groups. This pattern supports previous findings that sex-specific vascular remodeling is influenced by hormonal [24] and genetic factors [25]. IMT progressively increased with age and became more pronounced after the age of 60 years. By the age of 60–64 years, the IMT of the carotid bulb exceeded 1.5 mm in both males and females, while the ICA also reached or surpassed the 1.5 mm threshold starting at the 75–79 age group in males and the 80–84 age group in females. In contrast, the mean IMT in the CCA remained below 1.5 mm across all age groups. These findings emphasize the distinct segmental differences in IMT progression, highlighting the bulb as the segment most prone to thickening with age and the variability in age-related remodeling patterns across carotid segments.

### 4.2. Associations Between IMT and CV Risk Factors

HTN and DM demonstrated the strongest associations with increased carotid IMT in regression analyses, particularly in the CCA and carotid bulb, underscoring their significant roles in vascular remodeling and early atherosclerotic changes. These results are consistent with previous studies demonstrating the influence of chronic HTN [26] and long-term hyperglycemia [27] on endothelial dysfunction [28], smooth muscle cell proliferation [29], and arterial wall thickening [30]. Dyslipidemia demonstrated weaker but significant associations with increased IMT, reflecting its modest contribution to vascular changes. However, given the large sample size, some statistically significant associations may not necessarily translate into strong clinical implications. Therefore, the careful interpretation of effect sizes is warranted. This finding is consistent with previous research that reported a significant correlation between dyslipidemia and increased IMT, particularly in individuals with multiple cardiovascular risk factors [10]. Dyslipidemia contributes to carotid IMT progression by promoting lipid accumulation, inflammatory responses, and foam cell formation within the arterial wall [31]. These findings underscore the importance of the aggressive management of these risk factors in preventing subclinical atherosclerosis.

### 4.3. Age- and Sex-Related Trends in Carotid IMT

Our study established segment-specific normative distributions of carotid IMT across various age groups and sexes in a healthy Korean population, offering reference values derived from individuals without major CV risk factors to serve as a benchmark for clinical practice. The mean CCA IMT ranged from 0.48 mm in males aged 20–24 years to 1.13 mm in the 75–79 years age group. The carotid bulb showed greater variability, with mean IMT levels reaching 1.2–1.4 mm in males and 1.1–1.3 mm in females when the CCA IMT was 0.8–0.9 mm. These findings align with those of previous research conducted in different ethnic groups, indicating similar age- and sex-related trends in carotid IMT. For instance, a comprehensive study combining data from 25 research centers worldwide reported that CCA IMT values increased steadily with age in both men and women [32]. In men aged 40–49 years, the 50th percentile (median) CCA IMT was approximately 0.50 mm, increasing to about 0.61 mm in the 60–69 age group. Similarly, in women, the median CCA IMT increased from around 0.48 mm in the 40–49 age group to 0.60 mm in the 60–69 age group. Our findings are also consistent with data from the Gutenberg Heart Study, which examined a community-based German population [33]. This study found that the median CCA IMT values increased with age in both sexes, with men generally exhibiting slightly higher IMT values than women. For example, in the 35–44 age group, the median IMT was 0.62 mm in men and 0.60 mm in women, increasing to 0.74 mm in men and 0.73 mm in women in the 65–74 age group. Slight variations in absolute IMT values between studies may be attributed to differences in the measurement techniques, population characteristics, and regional factors. Due to the small number of participants aged 80–84 years, the results in this age group should be interpreted with caution.

### 4.4. Limitations

Although this study offers robust findings, several limitations should be acknowledged. First, the cross-sectional design limits the ability to draw causal inferences about the relationship between IMT and cardiovascular risk factors, and the study lacks longitudinal outcome data to assess its predictive value for clinical events such as myocardial infarction or stroke. Second, although this study included a large cohort of South Korean adults, its findings may not be generalizable to other ethnicities or regions. Third, excluding poor-quality ultrasound images to ensure accuracy may have introduced a selection bias, potentially underestimating IMT variability in broader populations. Fourth, although comprehensive adjustments were made for confounding variables, unmeasured factors such as dietary patterns or genetic predispositions may still have influenced the results. Fifth, while statistically significant differences were observed between groups and in regression analyses, some effect sizes were relatively small, which may limit their clinical relevance. These findings highlight the importance of interpreting statistical significance within the broader context of cardiovascular risk assessment. Sixth, although our study defined dyslipidemia using both biochemical and self-reported criteria, we acknowledge that hypercholesterolemia and hypertriglyceridemia may have distinct effects on IMT. Future studies should explore these lipid subtypes separately to better understand their differential impact on vascular remodeling. Finally, the analysis of the normal IMT distribution focused solely on individuals without HTN, DM, or dyslipidemia. Other subclinical risk factors, such as inflammation or genetic predispositions, were not assessed, potentially underestimating IMT variability in ostensibly healthy populations.

## 5. Conclusions

This study provides an extensive evaluation of carotid IMT, offering updated segment-specific reference values and insights into its association with CV risk factors. These findings contribute to a better understanding of subclinical atherosclerosis, particularly in Asian populations. The data underscore the importance of segmental considerations and age- and sex-specific reference ranges in clinical and research applications. Future studies should prioritize longitudinal designs and explore the integration of carotid IMT into comprehensive CV risk prediction models.

## Figures and Tables

**Table 1:** Baseline characteristics stratified by carotid intima-media thickness across different carotid artery segments.

Variables	CCA Carotid		*p*-Value	Bulb Carotid		*p*-Value	ICA Carotid		*p*-Value
	<1.5 mm	≥1.5 mm		<1.5 mm	≥1.5 mm		<1.5 mm	≥1.5 mm	
*n*	85,164	987		67,568	7813		83,997	1127	
Age (years)	46.44 (9.47)	59.79 (10.11)	<0.001	45.25 (8.98)	54.98 (10.38)	<0.001	46.35 (9.41)	60.63 (10.00)	<0.001
Sex (Male, *n*, %)	59133 (69.43)	759 (76.90)	<0.001	45,725 (67.67)	5858 (74.98)	<0.001	58,145 (69.22)	918 (81.46)	<0.001
Body Mass Index (kg/m^2^)	24.62 (3.54)	24.87 (3.14)	0.027	24.56 (3.61)	24.70 (3.18)	0.001	24.61 (3.54)	24.79 (3.08)	0.097
Waist Circumference (cm)	85.24 (9.95)	86.62 (8.57)	<0.001	84.93 (10.13)	85.88 (9.02)	<0.001	85.21 (9.96)	87.08 (8.53)	<0.001
Weight (kg)	71.19 (13.69)	69.88 (12.42)	0.003	71.06 (13.90)	70.54 (12.57)	0.002	71.18 (13.72)	69.82 (11.66)	0.001
Systolic Blood Pressure (mmHg)	113.20 (12.93)	122.12 (14.64)	<0.001	112.35 (12.76)	118.29 (13.33)	<0.001	113.16 (12.93)	121.06 (13.90)	<0.001
Diastolic Blood Pressure (mmHg)	72.05 (8.97)	74.59 (9.46)	<0.001	71.61 (8.96)	74.20 (8.79)	<0.001	72.03 (8.98)	74.07 (9.06)	<0.001
NT-proBNP	34.50 (72.59)	136.55 (571.96)	<0.001	33.28 (81.50)	56.24 (187.73)	<0.001	34.69 (82.49)	96.19 (408.38)	<0.001
Glucose (mg/dL)	97.91 (16.34)	107.72 (27.15)	<0.001	97.16 (15.58)	102.01 (19.83)	<0.001	97.85 (16.30)	105.89 (23.54)	<0.001
Hemoglobin (g/dL)	14.53 (1.41)	14.45 (1.29)	0.078	14.50 (1.44)	14.50 (1.30)	0.682	14.53 (1.42)	14.54 (1.25)	0.792
HemoglobinA1c (%)	5.68 (0.61)	6.12 (0.97)	<0.001	5.64 (0.57)	5.89 (0.73)	<0.001	5.67 (0.60)	6.06 (0.83)	<0.001
Total Cholesterol (mg/dL)	198.82 (38.19)	187.79 (47.94)	<0.001	199.11 (37.23)	192.58 (43.44)	<0.001	198.90 (38.12)	184.16 (46.68)	<0.001
LDL-Cholesterol (mg/dL)	128.17 (35.75)	121.38 (44.91)	<0.001	128.21 (34.85)	124.36 (40.82)	<0.001	128.25 (35.70)	117.49 (43.47)	<0.001
HDL-Cholesterol (mg/dL)	59.25 (15.56)	54.03 (13.36)	<0.001	59.72 (15.78)	57.10 (14.40)	<0.001	59.29 (15.56)	54.40 (13.54)	<0.001
Triglycerides (mg/dL)	130.77 (87.77)	132.65 (97.96)	0.507	129.59 (88.41)	127.48 (80.28)	0.044	130.53 (87.52)	129.85 (90.26)	0.797
eGFR	97.63 (13.80)	85.27 (15.59)	<0.001	98.64 (13.67)	89.33 (13.92)	<0.001	97.68 (13.79)	84.75 (14.88)	<0.001
	96.95 (17.95)	87.54 (18.29)	<0.001	97.68 (18.08)	90.16 (16.58)	<0.001	96.97 (17.96)	87.35 (17.65)	<0.001
AST (IU/L)	23.97 (13.72)	25.74 (12.64)	<0.001	23.67 (13.37)	25.34 (16.24)	<0.001	23.93 (13.73)	26.63 (13.78)	<0.001
ALT (IU/L)	26.67 (20.44)	26.83 (18.34)	0.808	26.39 (20.63)	26.63 (18.73)	0.320	26.62 (20.45)	27.60 (18.44)	0.111
GGT (IU/L)	35.41 (43.73)	42.43 (87.50)	<0.001	34.65 (41.74)	36.93 (46.71)	<0.001	35.31 (43.49)	42.27 (89.99)	<0.001
Insulin (IU/mL)	8.20 (5.72)	7.42 (4.76)	<0.001	8.18 (5.65)	7.49 (6.17)	<0.001	8.19 (5.71)	7.47 (4.88)	<0.001
HOMA-IR	2.04 (1.67)	2.05 (1.67)	0.931	2.02 (1.65)	1.95 (1.85)	0.001	2.04 (1.67)	2.01 (1.55)	0.526
Uric Acid (mmol/L)	5.61 (1.44)	5.56 (1.33)	0.282	5.59 (1.45)	5.55 (1.37)	0.017	5.60 (1.44)	5.58 (1.35)	0.626
hsCRP (mg/L)	0.12 (0.32)	0.14 (0.51)	0.016	0.12 (0.33)	0.12 (0.34)	0.568	0.12 (0.32)	0.14 (0.53)	0.032
Lifestyle and Socioeconomic Variables									
Current Smoker (*n*, %)	13,754 (29.98)	202 (31.03)	0.591	10,563 (30.32)	1411 (29.37)	0.186	13,465 (29.90)	244 (31.73)	0.289
Alcohol Intake (g/day)	8.64 (14.49)	10.19 (15.45)	0.002	8.51 (14.34)	9.94 (16.46)	<0.001	8.61 (14.47)	10.83 (15.42)	<0.001
High Alcohol Intake (%)	1055 (1.37)	14 (1.62)	0.634	818 (1.34)	135 (1.92)	<0.001	1040 (1.37)	15 (1.49)	0.839
Alcohol Consumption (frequency, *n* (%)):			<0.001			<0.001			<0.001
0 times/week	7869 (10.20)	144 (16.61)		5981 (9.78)	915 (12.95)		7784 (10.23)	134 (13.32)	
1–2 times/week	57,763 (74.84)	551 (63.55)		46199 (75.53)	4874 (68.97)		56,973 (74.86)	676 (67.20)	
3–4 times/week	9044 (11.72)	112 (12.92)		7103 (11.61)	932 (13.19)		8910 (11.71)	133 (13.22)	
≥5 times/week	2502 (3.24)	60 (6.92)		1885 (3.08)	346 (4.90)		2444 (3.21)	63 (6.26)	
Vigorous Exercise (≥5 times/week, *n* (%))	4288 (5.05)	86 (8.83)	<0.001	3341 (4.96)	483 (6.23)	<0.001	4232 (5.05)	80 (7.19)	0.002
Physical Activity Level (IPAQ %):			<0.001			<0.001			<0.001
Sedentary	28,693 (33.75)	262 (26.82)		22,978 (34.06)	2282 (29.35)		28,273 (33.72)	315 (28.20)	
Mild	37,761 (44.42)	420 (42.99)		30156 (44.70)	3433 (44.15)		37,299 (44.48)	482 (43.15)	
HEPA (Health-Enhancing Physical Activity)	18,560 (21.83)	295 (30.19)		14329 (21.24)	2060 (26.50)		18,276 (21.80)	320 (28.65)	
Health Conditions									
Hypertension (*n*, %)	24,264 (28.50)	608 (61.66)	<0.001	17,284 (25.59)	3550 (45.48)	<0.001	23,703 (28.23)	673 (59.77)	<0.001
Diabetes Mellitus (*n*, %)	9659 (11.34)	313 (31.71)	<0.001	6685 (9.89)	1597 (20.44)	<0.001	9432 (11.23)	323 (28.66)	<0.001
Dyslipidemia (*n*, %)	51,112 (60.02)	638 (64.64)	0.004	39,427 (58.35)	4780 (61.18)	<0.001	50,337 (59.93)	673 (59.72)	0.910

Abbreviations: NT-proBNP, N-terminal pro-B-type natriuretic peptide; HbA1c, Hemoglobin A1c; LDL-Cholesterol, Low-density lipoprotein cholesterol; HDL-Cholesterol, High-density lipoprotein cholesterol; eGFR, Estimated glomerular filtration rate; AST, Aspartate aminotransferase; ALT, Alanine aminotransferase; GGT, Gamma-glutamyl transferase; HOMA-IR, Homeostatic model assessment of insulin resistance; hsCRP, High-sensitivity C-reactive protein; IPAQ, International Physical Activity Questionnaire; HEPA, Health-Enhancing Physical Activity.

**Table 2:** Association between CVD history and carotid IMT values.

Outcome Variable	Predictor Variable	Crude	Model 1 *	Model 2 †	Model 3 ‡
		Beta (95% CI), *p*-Value	Beta (95% CI), *p*-Value	Beta (95% CI), *p*-Value	Beta (95% CI), *p*-Value
Common Carotid Artery	HTN	0.11 (0.10–0.11), <0.001	0.04 (0.04–0.04), <0.001	0.03 (0.02–0.04), <0.001	0.03 (0.02–0.04), <0.001
	DM	0.12 (0.11–0.12), <0.001	0.04 (0.04–0.05), <0.001	0.04 (0.02–0.05), <0.001	0.03 (0.02–0.05), <0.001
	Dyslipidemia	0.03 (0.03–0.03), <0.001	0.02 (0.02–0.02), <0.001	0.01 (0.00–0.02), 0.007	0.01 (0.00–0.02), 0.004
Carotid Bulb	HTN	0.18 (0.18–0.19), <0.001	0.05 (0.05–0.06), <0.001	0.05 (0.03–0.07), <0.001	0.05 (0.03–0.07), <0.001
	DM	0.19 (0.18–0.20), <0.001	0.04 (0.03–0.05), <0.001	0.03 (0.01–0.06), 0.014	0.03 (0.01–0.06), 0.014
	Dyslipidemia	0.03 (0.02–0.04), <0.001	0.01 (0.00–0.02), <0.001	0.02 (0.00–0.03), 0.029	0.02 (0.00–0.04), 0.013
Internal Carotid Artery	HTN	0.10 (0.09–0.10), <0.001	0.03 (0.03–0.04), <0.001	0.03 (0.02–0.04), <0.001	0.03 (0.02–0.04), <0.001
	DM	0.10 (0.10–0.11), <0.001	0.03 (0.03–0.04), <0.001	0.03 (0.02–0.04), <0.001	0.03 (0.02–0.04), <0.001
	Dyslipidemia	0.03 (0.02–0.03), <0.001	0.02 (0.01–0.02), <0.001	0.01 (0.00–0.02), 0.002	0.01 (0.01–0.02), <0.001

Abbreviations: IMT, intima-media thickness; CVD, cardiovascular disease; HTN, hypertension; DM, diabetes mellitus; BMI, body mass index; NT-proBNP, N-terminal pro-B-type natriuretic peptide; eGFR, estimated glomerular filtration rate; HOMA-IR, homeostatic model assessment of insulin resistance. Model Adjustments: * Model 1, adjusted for age and sex; † Model 2, adjusted for Model 1 + BMI, NT-proBNP, eGFR, and HOMA-IR; ‡ Model 3, adjusted for Model 2 + vigorous exercise and physical activity level.

**Table 3:** Association between CVD history and abnormality of carotid IMT (cut-off: 1.5 mm).

Outcome Variable	Predictor Variable	Crude	Model 1 *	Model 2 †	Model 3 ‡
		Odds Ratio (95% CI), *p*-Value	Odds Ratio (95% CI), *p*-Value	Odds Ratio (95% CI), *p*-Value	Odds Ratio (95% CI), *p*-Value
Common Carotid Artery	HTN	4.04 (3.55–4.59), <0.001	2.07 (1.81–2.37), <0.001	1.60 (1.11–2.30), 0.012	1.60 (1.11–2.31), 0.011
	DM	3.63 (3.17–4.16), <0.001	1.81 (1.57–2.09), <0.001	2.13 (1.48–3.08), <0.001	2.13 (1.48–3.07), <0.001
	Dyslipidemia	1.22 (1.07–1.39), 0.003	1.79 (1.56–2.06), <0.001	1.59 (1.11–2.27), 0.011	1.59 (1.12–2.28), 0.011
Carotid Bulb	HTN	2.43 (2.31–2.54), <0.001	1.39 (1.32–1.46), <0.001	1.38 (1.19–1.61), <0.001	1.38 (1.19–1.61), <0.001
	DM	2.34 (2.20–2.49), <0.001	1.28 (1.19–1.36), <0.001	1.26 (1.05–1.51), 0.014	1.27 (1.05–1.52), 0.012
	Dyslipidemia	1.12 (1.07–1.18), <0.001	1.27 (1.20–1.34), <0.001	1.24 (1.07–1.43), 0.004	1.25 (1.08–1.44), 0.003
Internal Carotid Artery	HTN	3.78 (3.35–4.26), <0.001	1.82 (1.60–2.06), <0.001	1.67 (1.19–2.34), 0.003	1.64 (1.17–2.31), 0.004
	DM	3.18 (2.79–3.62), <0.001	1.47 (1.28–1.69), <0.001	1.72 (1.21–2.44), 0.002	1.78 (1.25–2.52), 0.001
	Dyslipidemia	0.99 (0.88–1.12), 0.886	1.51 (1.32–1.72), <0.001	1.39 (1.00–1.93), 0.051	1.42 (1.02–1.98), 0.038

Abbreviations: IMT, intima-media thickness; CVD, cardiovascular disease; HTN, hypertension; DM, diabetes mellitus; BMI, body mass index; NT-proBNP, N-terminal pro-B-type natriuretic peptide; eGFR, estimated glomerular filtration rate; HOMA-IR, homeostatic model assessment of insulin resistance. Model Adjustments: * Model 1, adjusted for age and sex; † Model 2, adjusted for Model 1 + BMI, NT-proBNP, eGFR, and HOMA-IR; ‡ Model 3, adjusted for Model 2 + vigorous exercise and physical activity level.

**Table 4:** Distribution of IMT according to 5 year age group in male without history of DM, HTN and dyslipdemia.

Age Group (Years)	*n*	CCA		Bulb		ICA	
		Mean (SD)	Median (IQR)	Mean (SD)	Median (IQR)	Mean (SD)	Median (IQR)
20–24	79	0.48 (0.06)	0.47 (0.07)	0.53 (0.08)	0.53 (0.1)	0.46 (0.06)	0.47 (0.09)
25–29	666	0.50 (0.07)	0.49 (0.09)	0.59 (0.15)	0.56 (0.12)	0.48 (0.07)	0.48 (0.09)
30–34	2191	0.52 (0.07)	0.50 (0.08)	0.64 (0.19)	0.60 (0.14)	0.50 (0.08)	0.49 (0.09)
35–39	3079	0.55 (0.09)	0.53 (0.10)	0.74 (0.28)	0.66 (0.20)	0.54 (0.11)	0.52 (0.11)
40–44	3370	0.57 (0.11)	0.55 (0.11)	0.80 (0.31)	0.70 (0.25)	0.56 (0.13)	0.54 (0.12)
45–49	2048	0.62 (0.14)	0.59 (0.12)	0.92 (0.39)	0.76 (0.44)	0.60 (0.14)	0.57 (0.12)
50–54	1274	0.67 (0.18)	0.62 (0.17)	1.03 (0.45)	0.88 (0.55)	0.65 (0.20)	0.60 (0.16)
55–59	644	0.77 (0.28)	0.68 (0.24)	1.18 (0.50)	1.07 (0.68)	0.75 (0.31)	0.66 (0.25)
60–64	400	0.83 (0.27)	0.74 (0.30)	1.33 (0.57)	1.23 (0.74)	0.81 (0.34)	0.70 (0.28)
65–69	230	0.91 (0.26)	0.86 (0.36)	1.40 (0.54)	1.32 (0.66)	0.92 (0.42)	0.81 (0.42)
70–74	130	0.96 (0.30)	0.88 (0.35)	1.47 (0.61)	1.34 (0.80)	0.92 (0.35)	0.82 (0.37)
75–79	52	1.13 (0.53)	1.04 (0.44)	1.74 (0.80)	1.60 (1.03)	1.11 (0.65)	0.88 (0.65)
80–84	8	0.95 (0.18)	0.92 (0.35)	1.40 (0.47)	1.36 (0.69)	1.09 (0.58)	0.86 (0.35)

Abbreviations: IMT, intima-media thickness; CCA, common carotid artery; ICA, internal carotid artery; Bulb, carotid bulb; DM, diabetes mellitus; HTN, hypertension; SD, standard deviation; IQR, interquartile range.

**Table 5:** Distribution of IMT according to 5 year age group in female without history of DM, HTN and dyslipdemia.

Age Group (Years)	*n*	CCA		Bulb		ICA	
		Mean (SD)	Median (IQR)	Mean (SD)	Median (IQR)	Mean (SD)	Median (IQR)
20–24	33	0.43 (0.05)	0.43 (0.07)	0.51 (0.10)	0.49 (0.15)	0.42 (0.06)	0.43 (0.07)
25–29	415	0.47 (0.05)	0.47 (0.07)	0.54 (0.10)	0.53 (0.10)	0.45 (0.06)	0.44 (0.07)
30–34	1303	0.49 (0.05)	0.48 (0.07)	0.58 (0.14)	0.56 (0.12)	0.47 (0.07)	0.46 (0.08)
35–39	1940	0.52 (0.08)	0.51 (0.08)	0.66 (0.21)	0.60 (0.15)	0.50 (0.10)	0.49 (0.10)
40–44	2615	0.55 (0.08)	0.53 (0.09)	0.71 (0.25)	0.64 (0.19)	0.52 (0.10)	0.50 (0.10)
45–49	1817	0.59 (0.10)	0.57 (0.10)	0.81 (0.30)	0.71 (0.29)	0.56 (0.12)	0.54 (0.13)
50–54	914	0.63 (0.12)	0.61 (0.13)	0.93 (0.36)	0.82 (0.49)	0.60 (0.15)	0.57 (0.15)
55–59	594	0.70 (0.19)	0.65 (0.16)	1.05 (0.40)	0.95 (0.59)	0.64 (0.17)	0.59 (0.18)
60–64	529	0.73 (0.18)	0.68 (0.16)	1.13 (0.45)	1.04 (0.58)	0.67 (0.19)	0.61 (0.18)
65–69	330	0.82 (0.26)	0.73 (0.24)	1.23 (0.48)	1.16 (0.74)	0.73 (0.26)	0.65 (0.23)
70–74	117	0.85 (0.24)	0.76 (0.29)	1.17 (0.46)	1.10 (0.50)	0.75 (0.23)	0.68 (0.23)
75–79	35	0.91 (0.20)	0.92 (0.29)	1.58 (0.60)	1.44 (0.83)	0.78 (0.15)	0.76 (0.21)
80–84	5	0.94 (0.25)	0.96 (0.29)	1.42 (0.79)	1.37 (1.07)	0.72 (0.21)	0.62 (0.24)

Abbreviations: IMT, intima-media thickness; CCA, common carotid artery; ICA, internal carotid artery; Bulb, carotid bulb; DM, diabetes mellitus; HTN, hypertension; SD, standard deviation; IQR, interquartile range.

## Data Availability

The data supporting the findings of this study are available from the corresponding author upon reasonable request.

## Supplementary Materials

The following supporting information can be downloaded at: https://www.mdpi.com/article/10.3390/jcm14061918/s1, Table S1: Repeatability and reproducibility of carotid intima-media thickness measurements across different anatomical locations.
