# Supplementary material for: Segment-Specific Analysis of Carotid Intima-Media Thickness and Its Association with Cardiovascular Risk Factors in a Large Healthy Cohort
Source: J Clin Med. 2025 Mar 12;14(6):1918. doi: 10.3390/jcm14061918 (PMC11943414; doi:10.3390/jcm14061918)
Supplement: Supplementary file 1 [file jcm-14-01918-s001.zip › jcm-3517768-supplementary.pdf]

Supplementary Table S1. Repeatability and reproducibility of carotid intima-media thickness measurements across different anatomical locations.

Location	N	ICC	CV	Bias	LOA Lower	LOA Upper
CCA Rt.	9113	0.843 (0.829-0.856)	8.52% (8.36%-.70%)	-0.0253 (-0.0282-0.0224)	-0.2981 (-0.3030-0.2932)	0.2475 (0.2427-0.2524)
CCALt.	9060	0.864 (0.851-0.875)	8.54% (8.37%-.73%)	-0.0275 (-0.0306-0.0244)	-0.3227 (-0.3280-0.3174)	0.2676 (0.2623-0.2729)
Bulb Rt.	7618	0.862 (0.852-0.871)	14.13% (13.84%-4.40%)	-0.0460 (-0.0528-0.0393)	-0.6356 (-0.6471-0.6240)	0.5435 (0.5320-0.5551)
Bulb Lt.	7525	0.860 (0.851-0.869)	13.71% (13.43%-4.05%)	-0.0468 (-0.0539-0.0396)	-0.6640 (-0.6762-0.6518)	0.5705 (0.5583-0.5826)
ICA Rt.	8880	0.799 (0.785-0.811)	15.51% (15.20%-5.78%)	-0.0365 (-0.0412-0.0317)	-0.4856 (-0.4937-0.4774)	0.4127 (0.4045-0.4208)
ICA Lt.	8851	0.807 (0.794-0.819)	15.27% (14.96%-5.58%)	-0.0388 (-0.0439-0.0336)	-0.5210 (-0.5297-0.5122)	0.4434 (0.4347-0.4522)

ICC, intra-class correlation coefficient; CV, coefficient of variation; LOA, limits of agreement; CCA, common carotid artery; ICA, internal carotid artery.

* This analysis was performed using the full dataset, including multiple follow-up carotid ultrasound measurements from the same individuals, ensuring a comprehensive assessment of measurement repeatability and reproducibility.
